# Bilateral traction method using a clip with thread for rectal endoscopic submucosal dissection

**DOI:** 10.1055/a-2496-2899

**Published:** 2024-12-17

**Authors:** Ryosuke Ikeda, Hiroaki Kaneko, Hiroki Sato, Aya Ikeda, Yoshihiro Goda, Kuniyasu Irie, Shin Maeda

**Affiliations:** 1 26438 Department of Gastroenterology, Yokohama City University School of Medicine Graduate School of Medicine, Yokohama, Japan

Countertraction during endoscopic submucosal dissection (ESD) is important for successful treatment. Recently, several traction methods for colorectal ESD have been reported 1 2 3 4 ; traction-assisted colonic (TAC)-ESD is a useful technique using a clip with thread 3 4 . Although TAC-ESD enables traction adjustment by pulling the thread, traction can only be performed in a specific direction. We report a case of a rectal tumor that was treated successfully by the adjustable bilateral traction (BLT) method using a clip with thread ( Video 1 ).

Successful treatment of rectal endoscopic submucosal dissection using bilateral traction method using a clip with thread.Video 1

An 86-year-old woman who presented with bloody stools underwent colonoscopy, which revealed a 25-mm protruded lesion in the rectum ( Fig. 1 ). The patient was referred to our hospital, and ESD was performed under conscious sedation.

**Fig. 1:**
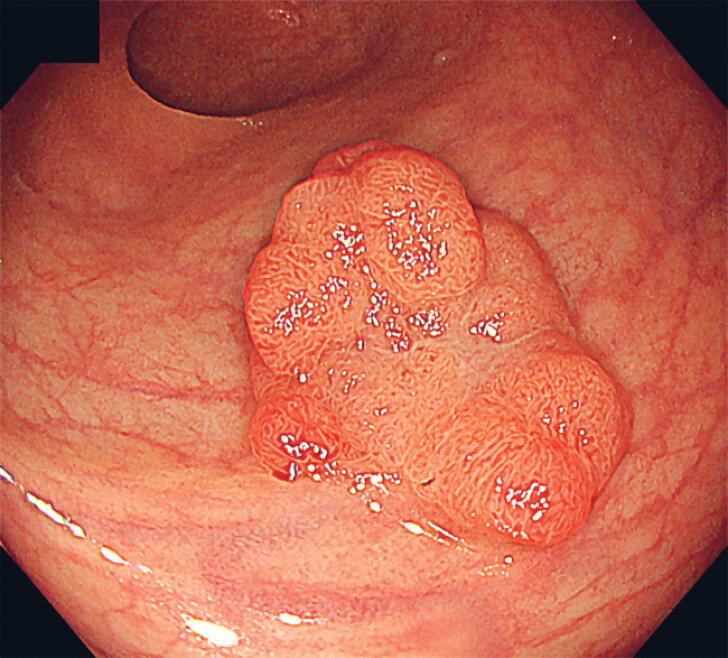
White-light imaging showed a 25-mm protruded reddish lesion in the rectum.

A mucosal incision was initiated from the oral side using a DualKnife (Olympus Medical Systems Co., Tokyo, Japan). After making a circumferential mucosal incision, we dissected the submucosal layer on the anal side to form a mucosal flap and attached the EZ clip (HX-610-090; Olympus Medical Systems Co.) with a double thread to the specimen for traction. Each part of the double thread was fixed to the left and right colonic walls, respectively, with reopenable clips (Sure clip; Micro-tech, Nanjing, China), allowing bilateral traction ( Fig. 2 ). Pulling the trochlear thread made it easier to maintain the view of the submucosal layer, and bilateral countertraction could be performed appropriately by pulling the threads. We first dissected the right edge of the specimen by pulling the left thread ( Fig. 3 **a, b** ), and then the left edge by pulling the right thread ( Fig. 4 **a, b** ). Subsequently, en bloc resection was completed and curative resection was achieved, with negative margins and no lymphovascular infiltration.

**Fig. 2:**
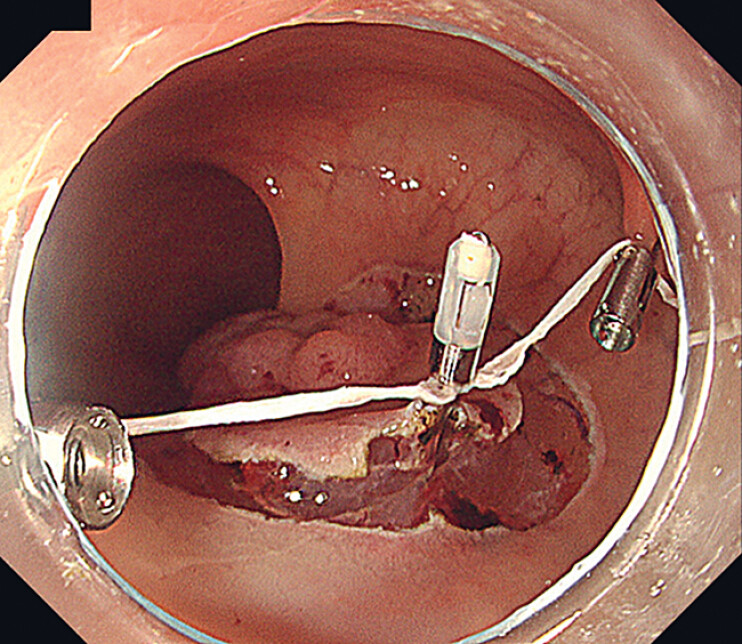
Bilateral traction was performed using a clip with thread. After attaching the clip with the double thread to the mucosal flap of the specimen, each part of the double thread was fixed to the left and right colonic walls, respectively.

**Fig. 3:**
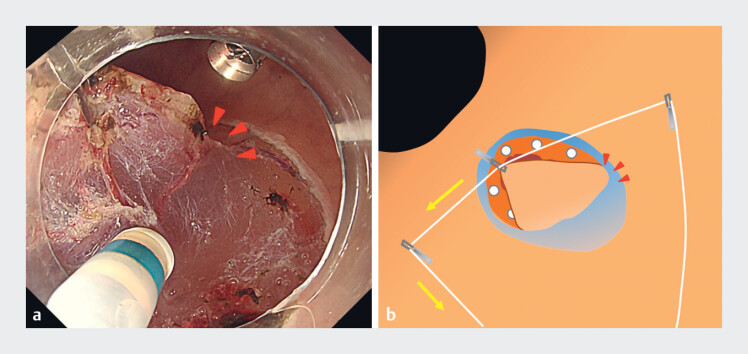
Applying traction during dissection. **a** Dissection of the right edge of the specimen. Countertraction to the left made it easier to maintain the resection view of the right edge (red arrowhead). **b** Schema: countertraction could be adjusted to the left direction by pulling the left trochlear thread.

**Fig. 4:**
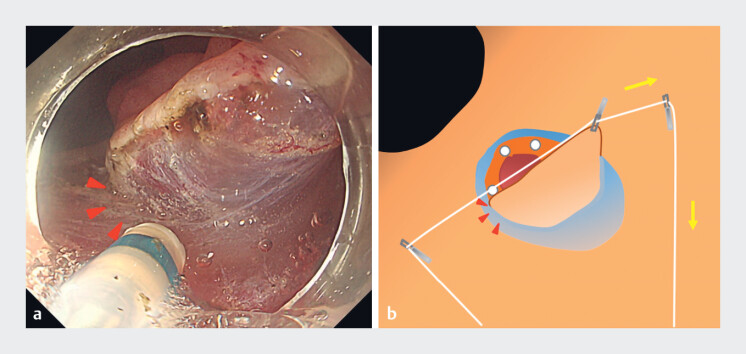
Applying traction during dissection. **a** Dissection of the left edge of the specimen. Countertraction to the right made it easier to maintain the resection view of the left edge (red arrowhead). **b** Schema: countertraction could be adjusted to the right direction by pulling the right trochlear thread.

The BLT method using a clip with thread permits adjustments to maintain the view required by the endoscopist, thus facilitating reliable en bloc resection.

Endoscopy_UCTN_Code_TTT_1AQ_2AD_3AD

## Notes

Endoscopy E-Videos https://eref.thieme.de/e-videos
*E-Videos* is an open access online section of the journal *Endoscopy* , reporting on interesting cases and new techniques in gastroenterological endoscopy. All papers include a high-quality video and are published with a Creative Commons CC-BY license. Endoscopy E-Videos qualify for HINARI discounts and waivers and eligibility is automatically checked during the submission process. We grant 100% waivers to articles whose corresponding authors are based in Group A countries and 50% waivers to those who are based in Group B countries as classified by Research4Life (see: https://www.research4life.org/access/eligibility/ ). This section has its own submission website at https://mc.manuscriptcentral.com/e-videos .
